# Validation of Reference Genes for Normalization Gene Expression in Reverse Transcription Quantitative PCR in Human Normal Thyroid and Goiter Tissue

**DOI:** 10.1155/2014/198582

**Published:** 2014-05-11

**Authors:** Raquel Weber, Ana Paula Santin Bertoni, Laura Walter Bessestil, Beatriz Maria de Azevedo Assis Brasil, llma Simoni Brum, Tania Weber Furlanetto

**Affiliations:** ^1^Programa de Pós Graduação em Medicina: Ciências Médicas, Universidade Federal do Rio Grande do Sul, Rua Ramiro Barcelos 2400, 90035-903 Porto Alegre, RS, Brazil; ^2^Programa de Pós Graduação em Ciências da Saúde, Universidade Federal de Ciências da Saúde Porto Alegre, Rua Sarmento Leite 245, 90050-170 Porto Alegre, RS, Brazil; ^3^Departamento de Fisiologia, Instituto de Ciências Básicas da Saúde, Universidade Federal do Rio Grande do Sul, Rua Sarmento Leite 500, 90050-170 Porto Alegre, RS, Brazil; ^4^Serviço de Patologia, Hospital de Clínicas de Porto Alegre, Rua Ramiro Barcelos 2350, 90035-903 Porto Alegre, RS, Brazil

## Abstract

Reverse transcription quantitative polymerase chain reaction (RT-qPCR) has been recognized as the most accurate method for quantifying mRNA transcripts, but normalization of samples is a prerequisite for correct data interpretation. So, this study aimed to evaluate the most stable reference gene for RT-qPCR in human normal thyroid and goiter tissues. Beta-actin (ACTB); glyceraldehyde-3-phosphate dehydrogenase (GAPDH); succinate dehydrogenase, subunit A, flavoprotein (Fp) (SDHA); hypoxanthine phosphoribosyltransferase I (HPRTI); tyrosine 3-monooxygenase/tryptophan 5-monooxygenase activation protein, zeta polypeptide (YWHAZ); and beta-2-microglobulin (B2M) were evaluated in 14 thyroid tissue samples (7 normal and 7 goiter tissues) by RT-qPCR. The mean Cq and the maximum fold change (MFC) and NormFinder software were used to assess the stability of the genes. As a result, ACTB gene was more stable than GAPDH, SDHA, HPRTI, YWHAZ, and B2M. In conclusion, ACTB could be used to normalize RT-qPCR data in normal thyroid and goiter tissues.

## 1. Introduction

Reverse transcription quantitative polymerase chain reaction (RT-qPCR) has been recognized as the most accurate, sensitive, and easy method for quantifying mRNA transcripts in biological samples [1, 2]. Nevertheless, normalization of samples is a prerequisite for correct data interpretation, since its accuracy is significantly affected by sample quality, reagent, and operator technique [3].

Cell number, quantification of rRNA or total RNA, and reference genes are some standardization methods that have been proposed so far [2, 4, 5]. Normalization with another gene, which is assumed to have stable expression, is the method of choice because this reference gene is exposed to the same preparation steps as the gene of interest [6]. Therefore, a full procedure control is obtained during the whole assay.

A good reference gene needs to present low variation in its expression in the different samples which would be evaluated and demonstrated to be minimally regulated during the individual experiment or pathological condition. Therefore, there is a need to validate genes to allow the accuracy of RNA transcription analysis [3, 6]. Nowadays, there is emerging evidence that reference genes could vary their expression in different cellular development stages and in other conditions [5, 7, 8].

The thyroid normal gland is a fairly homogenous structure, but in goiter a genetic heterogeneity is described in follicular cells; some nodules have a polyclonal origin and others a monoclonal origin. These changes may be related to mutations in oncogenes which do not produce malignancy per se but would alter growth and function [14, 15]. These alterations could modify the expression of many thyroid genes, including the ones usually described as reference genes, so the evaluation of candidates to normalize RT-qPCR is necessary.

Little is known about the expression stability of reference genes in thyroid cells or tissue, as shown in Table 1.

Considering the species, there are many differences in reference genes stability evaluated in these studies. Although in human tissues the same software was used to analyze data, differences in RT-qPCR and cells status could have contributed to these findings. In addition, a stable expression of a reference gene in one tumor type does not predict a stable expression in another tumor type [7]. In this way, there is no universal reference gene being necessary to validate potential reference genes for any experimental condition. So, this study aimed to evaluate the most stable reference gene for RT-qPCR in human normal thyroid and goiter tissues.

## 2. Material and Methods

### 2.1. Tissue Acquisition

Normal thyroid and goiter tissues were obtained from patients who underwent total thyroidectomy as part of treatment for differentiated thyroid cancer in the Hospital de Clínicas de Porto Alegre (HCPA). After the evaluation of macroscopic and frozen sections of surgical specimens by two pathologists, samples of normal thyroid or goiter tissue were frozen in liquid nitrogen and stored at −80°C until further processing. The pattern used to confirm the presence of goiter was a well-defined fibrous capsule with a mixture of macrofollicles and microfollicles and, in some cases, some degenerative changes such as fibrosis and hemorrhage [16]. This study was approved by the Ethics Committee of HCPA (GPPG: 12-0272).

### 2.2. Nucleic Acid Extraction and Reverse Transcription

Normal thyroid and goiter tissue were homogenized mechanically with Omnimix for 30 seconds in Trizol (Invitrogen, Life Technologies, Carlsbad, CA, USA), and total RNA was extracted with the same commercial kit, according to the manufacturer's protocol. The RNA concentration and purity were assessed with the NanoDrop 1000 Spectrophotometer (Thermo Fisher Scientific, Wilmington, DE, USA). Samples were then stored at −80°C. 1 *μ*g total RNA was reverse-transcribed to produce cDNA using random and oligo-dT primers and Superscript II reverse transcriptase (Invitrogen Life Technologies) in total reaction volumes of 20 *μ*L. All cDNA samples were diluted 10-fold with diethyl pyrocarbonate- (DEPC-) treated water and stored at −20°C.

### 2.3. Gene Selection and Quantitative PCR

Six genes commonly used as reference in RT-qPCR gene expression experiments were selected (Table 1): beta-actin (ACTB, related to cell structure); glyceraldehyde-3-phosphate dehydrogenase (GAPDH, related to carbohydrate metabolism); succinate dehydrogenase, subunit A, flavoprotein (Fp) (SDHA, related to energy metabolism); hypoxanthine phosphoribosyltransferase I (HPRTI, related to nucleotide metabolism); tyrosine 3-monooxygenase/tryptophan 5-monooxygenase activation protein, zeta polypeptide (YWHAZ, related to cell growth and death); and beta-2-microglobulin (B2M, related to major histocompatibility complex). Primers sequence shown in Table 2 was described previously [17]. Quantification of amplified samples was performed based on a standard curve with five successive tenfold dilution points of a pool of cDNA samples.

RT-qPCR was performed using Applied Biosystems StepOne Real-Time PCR System using Kit Platinum SYBR Green qPCR SuperMix-UDG (Invitrogen Life Technologies, Carlsbad, CA, USA). The RT-qPCR was performed with an initial denaturation (10 min at 95°C), followed by 40 cycles of denaturation (15 s at 95°C), annealing, and extension (45 s at 60°C). Melting curve was acquired to ensure that a single product was amplified in each reaction.

### 2.4. Statistics

To evaluate the stability of the candidate reference genes in thyroid normal and goiter tissues, the NormFinder algorithm was used [18]. In addition, the analysis of raw quantification cycle (Cq) values of each gene was used to evaluate their stability. Mean Cq values, standard deviation (SD), coefficient of variation (CV), and maximum fold change (MFC, the ratio of the maximum and minimum values observed within the dataset) were calculated.

## 3. Results

**Figure 1:**
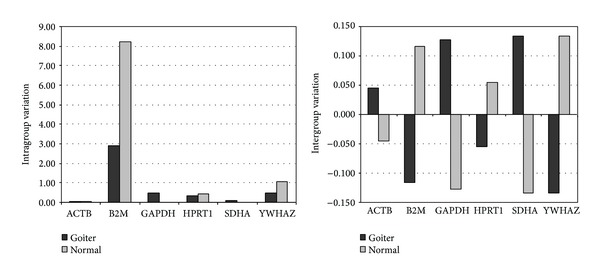
Intra- and intergroup variation of six candidate reference genes in goiter and normal thyroid tissues samples.

Six reference genes were amplified in 14 thyroid samples: 7 normal and 7 goiter. All RT-qPCR assays produced a single peak in the melting curve. The mean Cq cycle values, SD, CV, and MFC obtained for each gene in goiter and normal thyroid are described in Table 3. Analyzing these data, none of the genes had a constant expression. Moreover, B2M showed higher variability in Cq values. Thereby, we proceeded the analysis of quantification data using the NormFinder algorithm. The values of stability of the candidate genes obtained from the NormFinder analysis are shown in Table 4. The most stable genes were ACTB for goiter and GAPDH for normal thyroid tissue. When all samples were analyzed together, the most stable genes were SDHA and ACTB. Stability values and intra/intergroup variations are shown, respectively, in Table 5 and Figure 1.

## 4. Discussion

In this study, we evaluated 6 reference genes in 14 thyroid specimens (7 normal thyroid and 7 goiter) using RT-qPCR. The genes with the lowest variations in expression for normal samples, goiter, and all samples were, respectively, GAPDH, ACTB, SDHA and ACTB, when evaluated by the NormFinder.

According to de Jonge et al. [19], a good candidate gene should present a small coefficient of variation and a MFC < 2. Moreover, a mean expression level lower than the maximum expression level subtracted with 2 SD is a prerequisite for a candidate reference gene. By these criteria, only B2M and GAPDH genes could be used as internal control for goiter, and none of these genes could be used for normal thyroid, although all had MFC < 2.

The NormFinder algorithm was used to rank the candidate reference genes based on their stability values. The gene expression is more stable when this value is closer to zero. The stability value less than 0.15 is the cutoff for an acceptable reference gene [4]. Considering these criteria, none of the selected genes could be used to normalize RT-qPCR data for goiter tissues, and GAPDH and SDHA could be used for normal thyroid. When all data were analyzed together, SDHA and ACTB genes had the lowest stability values, respectively, 0.076 and 0.082. We suggest using ACTB as reference gene, when comparing gene expression in normal thyroid and goiter, because this gene had the lowest intra- and intergroup variations. Our results differ somewhat from those of Chantawibul et al. [10] who found the highest stability value for HPRT1, while the GAPDH had the lowest stability value. These differences may partly be explained by the sample selection, which included goiters, adenomas, carcinomas, and lymphocytic thyroiditis.

ACTB mRNA is expressed at moderately abundant levels in most cell types and its protein plays a key role in the cytoskeleton maintenance. Recently, some studies have suggested that this gene does not satisfy the basic requirements for application as internal control due to change in expression under various biomedical stimuli [20, 21]. Nevertheless, in normal thyroid cells, ACTB expression appeared to be stable in response to different experimental treatments [9]. Other studies have demonstrated that ACTB can be used as reference gene [22–24] for different cell types.

One limitation of this study is the small number of samples of goiter; nevertheless, all samples were evaluated by two pathologists, who used the same criteria to define goiter.

In conclusion, the results of the present study suggest that ACTB gene is more stable than SDHA, GAPDH, HPRTI, YWHAZ, and B2M when evaluating human normal thyroid and goiter together.

## Figures and Tables

**Table 1:** Stability of genes candidates to normalize RT-qPCR for thyroid derived biological material.

Study	Material	Reference gene stability	Method
Santin et al., 2013 [9]	Human normal cells	ACTB>TBP>GAPDH>B2M	NormFinder
Chantawibul et al., 2012 [10]	Human tissues*	GAPDH>ACTB>B2M>RPLIA>HPRT1	NormFinder
Lecchi et al., 2012 [11]	Bovine tissues, including thyroid	SF3A1=HBMS>ACTB	geNorm
		HMBS>ACTB>GAPDH	NormFinder
Li et al., 2011 [12]	Porcine thyroid tissue	18S rRNA>Ubiquitin>Histone H3>ACTB	geNorm
		18S rRNA>Ubiquitin>Histone H3>ACTB>GAPDH	NormFinder
Lisowski et al., 2008 [13]	Bovine thyroid tissue	TBP=HPRT1>YWHAZ>ACTB	geNorm

*13 goiters, 6 adenomas, 4 carcinomas (2 follicular + 2 papillary), and 2 lymphocytic thyroiditis.

**Table 2:** Primers sequence, product length, and NCBI reference sequence for reference genes by RT-qPCR.

Gene symbol	Name	Forward and reverse primers	Product length (bp)	NCBI reference sequence
ACTB	Beta-actin	5′-CTGGAACGGTGAAGGTGACA-3′	140	NM_001101.3
		5′-AAGGGACTTCCTGTAACAATGCA-3′		

B2M	Beta-2-microglobulin	5′-CTATCCAGCGTACTCCAAAG-3′	165	NM_004048.2
		5′-ACAAGTCTGAATGCTCCACT-3′		

GAPDH	Glyceraldehyde-3-phosphate dehydrogenase	5′-CTTTGTCAAGCTCATTTCCTGG-3′	133	NM_002046.3
		5′-TCTTCCTCTTGTGCTCTTGC-3′		

HPRT1	Hypoxanthine phosphoribosyltransferase 1	5′-AGATGGTCAAGGTCGCAAG-3′	128	NM_000194.2
		5′-GTATTCATTATAGTCAAGGGCATATCC-3′		

SDHA	Succinate dehydrogenase complex, subunit A, flavoprotein (Fp)	5′-TGGTTGTCTTTGGTCGGG-3′	85	NM_004168.2
		5′-GCGTTTGGTTTAATTGGAGGG-3′		

YWHAZ	Tyrosine 3-monooxygenase/tryptophan 5-monooxygenase activation protein, zeta polypeptide	5′-CAACACATCCTATCAGACTGGG-3′ 5′-AATGTATCAAGTTCAGCAATGGC-3′	133	NM_001135699.1

bp: base pair. Modified from Souza et al., 2012 [17].

**Table 3:** Dispersion data of raw Cq values for candidate reference genes in goiter and normal thyroid tissue.

Tissue	Symbol gene	Mean Cq	SD	CV (%)	Minimum	Maximum	MFC
Goiter	ACTB	21.88	1.78	8.1	19.78	25.00	1.26
	B2M	20.83	2.59	9.3	18.10	26.11	1.44
	GAPDH	21.18	1.95	9.2	19.54	25.60	1.31
	HPRT1	30.10	3.10	10.3	25.48	35.24	1.38
	SDHA	31.17	2.21	7.1	29.51	34.56	1.17
	YWHAY	27.48	2.47	9.0	22.71	30.93	1.36

Normal	ACTB	23.56	1.92	8.1	19.99	25.69	1.29
	B2M	24.63	5.08	20.6	18.53	32.65	1.76
	GAPDH	22.84	1.78	7.8	20.54	26.20	1.28
	HPRT1	32.12	3.06	9.5	25.65	35.66	1.39
	SDHA	33.94	3.20	9.4	29.54	39.39	1.33
	YWHAY	26.29	2.35	8.9	22.83	29.25	1.28

SD: standard deviation; CV: coefficient of variation; and MFC: maximum fold change (the ratio of the maximum and minimum values).

**Table 4:** Stability values of reference genes candidates calculated by the NormFinder software for each tissue.

Tissue	Symbol gene	Stability value
Goiter	ACTB	0.228
	SDHA	0.298
	HPRT1	0.567
	GAPDH	0.685
	YWHAZ	0.688
	B2M	1.711

Normal	GAPDH	0.114
	SDHA	0.114
	ACTB	0.221
	HPRT1	0.643
	YWHAZ	1.026
	B2M	2.867

**Table 5:** Stability values of reference candidate genes calculated by the NormFinder software for all tissues.

Symbol gene	Stability value
SDHA	0.076
ACTB	0.082
GAPDH	0.150
HPRT1	0.221
YWHAZ	0.311
B2M	0.830
